# FeNi-Based Aerogels Containing FeNi_3_ Nanoclusters Embedded with a Crystalline–Amorphous Heterojunction as High-Efficiency Oxygen Evolution Catalysts

**DOI:** 10.3390/molecules29225429

**Published:** 2024-11-18

**Authors:** Tao Li, Jiahui Chen, Zihao Song, Shujie Zhong, Wei Feng

**Affiliations:** School of Mechanical Engineering, Chengdu University, Chengdu 610106, China; cjh1245241937@163.com (J.C.); 17380220470@163.com (Z.S.); 18202800348@163.com (S.Z.)

**Keywords:** nanoclusters embedded, crystalline–amorphous heterojunction, FeNi-based, amorphous aerogels

## Abstract

In green hydrogen production via water electrolysis, catalysts with multiscale nanostructures synthesized by compositing micro-heterojunctions and nanoporous structures exhibit excellent electrocatalytic oxygen evolution reaction (OER) performance. Moreover, they are the most promising non-noble metal catalysts. Herein, FeNi-based aerogels with a three-dimensional nanoporous structure and amorphous matrix embedded with FeNi_3_ nanoclusters were synthesized via wet chemical reduction coprecipitation. The FeNi_3_ nanoclusters and the FeNi-based amorphous matrix formed a crystalline–amorphous heterojunction. These aerogels exhibited excellent OER performance and electrocatalytic stability in alkaline electrolytes. In 1 mol/L of KOH electrolyte, the as-synthesized aerogel exhibited an overpotential of 262 mV at a current density of 20 mA cm^−2^ with a Tafel slope of only 46 mV dec^−1^. It also demonstrated excellent stability during a 12 h chronopotentiometry test.

## 1. Introduction

Water electrolysis is a sustainable approach for green hydrogen production. Moreover, the synthesis of efficient catalysts is crucial for overcoming the kinetic barrier of reaction and improving the hydrogen production efficiency. Noble metal catalysts such as platinum, ruthenium, and iridium exhibit excellent catalytic performance; however, their high costs, limited availability, and poor stability significantly restrict their applications in the field of oxygen evolution reaction (OER) [1,2]. Some researchers have addressed these issues by introducing small amounts of noble metal elements into low-cost transition metal catalysts, but the preparation steps tend to be complicated [3]. Therefore, the development of catalysts that are simultaneously simple to prepare, cost-effective, and highly efficient has become a major research focus. Among these, multielement transition metal-based catalysts have garnered research attention [4,5], including those based on nickel–iron, nickel–cobalt, nickel–iron–cobalt, high-entropy alloys and compounds, graphene, and supported by carbon nanotubes [6,7,8,9,10]. Oxygen evolution reaction (OER) catalysts based on nickel–iron alloys are one of the most promising non-noble metal catalysts; this is attributed to the coupling effect between Fe and Ni, which synergistically regulates the electron transfer rate, thereby enhancing OER performance [11]. The electrocatalytic oxygen evolution of nickel–iron-based bimetallic structures can be enhanced via nano structuring and multielement composition [12,13,14]. These catalysts generally exhibit an overpotential of ~300 mV at a current density of 10 mA cm^−2^, indicating that there is still a significant improvement.

Amorphous materials contain randomly arranged atoms, resulting in abundant distorted lattices and unsaturated bonds. These can generate various defect states and easily form numerous unsaturated coordination sites in such materials. These sites become electrochemically active reaction sites for the OER [15,16]. Although amorphous nanocrystals have relatively weak conductivity that affects the charge transfer kinetics, they offer higher structural flexibility than crystalline nanocrystals [17]. This allows for their rapid self-reconstruction during electrochemical activities to adapt to the OER demands, which further enhances the reaction performance [18]. Recently developed electrocatalytic materials with heterojunction interfaces have exhibited excellent electrocatalytic performance. Lin et al. [19] developed MoS_2_/NiS_2_ nanosheets loaded on a carbon paper, which exhibited an overpotential of 278 mV at a current density of 10 mA cm^−2^. Qiu et al. [20] used loaded Ce and Co on CuO via electrodeposition, thereby achieving a low overpotential of 265 mV at a current density of 10 mA cm^−2^. They used conventional two-phase synthesis methods, thus making the synthesis of metal nanoparticles with tightly bonded crystalline–amorphous (c–a) heterostructures difficult. As a result, loosely bonded interfaces were formed between the crystalline and amorphous phases of the catalysts, which increased the charge transfer resistance and reduced the OER performance. These issues can be resolved using in situ recombination techniques.

Aerogels have high porosity and a self-supporting three-dimensional (3D) framework structure; therefore, they provide more active sites for the OER. The interconnected 3D network considerably accelerates electron transfer; thus, aerogels exhibit superior catalytic performance in the OER [21,22,23]. Although many aerogels have exhibited excellent OER catalytic performance, only noble metal aerogels and carbon-based aerogels loaded with non-noble metals via complex synthesis processes have been studied. These synthesis processes are slow and technologically complex [24,25,26,27]. The easily accessible and technically simple synthesis of transition metal aerogels with crystalline–amorphous heterostructures has been scarcely studied. Herein, amorphous aerogels embedded with nanoclusters were designed using dimethylamine borane with mild reducibility as a reducing agent and employing wet chemical reduction coprecipitation. Additionally, FeNi-based nanocatalysts with FeNi_3_ nanoclusters embedded in the crystalline–amorphous heterostructure and a porous aerogel composite structure were successfully prepared. The unique organizational characteristics and excellent OER performance of the aerogel were studied, based on which transition metal-based aerogels with nanocluster-embedded and crystalline–amorphous heterostructures can be synthesized.

## 2. Materials and Methods

### 2.1. Materials

Nickel chloride hexahydrate (NiCl_2_·6H_2_O) was purchased from Shanghai Titan Technology Co., Ltd. (Shanghai, China). Iron chloride hexahydrate (FeCl_3_·6H_2_O) was acquired from Shanghai Titan Technology Co., Ltd. (Shanghai, China). Potassium hydroxide (KOH) was obtained from Shanghai Titan Technology Co., Ltd. (Shanghai, China). Dimethylamine borane (DMAB) complex was sourced from Shanghai Titan Technology Co., Ltd. (Shanghai, China). Anhydrous ethanol (≥99.7%) was purchased from Shanghai Titan Technology Co., Ltd. (Shanghai, China). Nafion solution (5 wt%) and carbon paper were procured from Shanghai Chu Xi Industrial Co., Ltd. (Shanghai, China). Deionized water was used in all the experiments. All reagents were used as received without further purification.

### 2.2. Synthesis of FeNi-Based Aerogels

**Figure 1:**
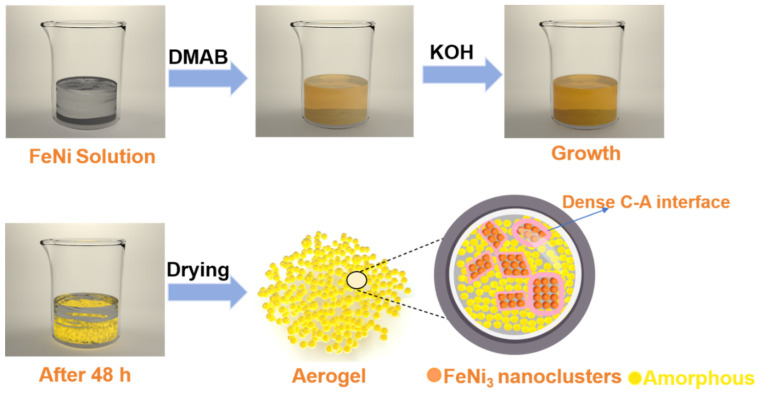
Schematic of the bottom-up synthesis of multimetallic FeNi aerogels.

A total of 50 mL of FeCl_3_·6H_2_O (2 mM) solution and 50 mL of NiCl_2_·6H_2_O (5 mM) solution were separately measured and mixed. The mixture was stirred to obtain 100 mL of FeCl_3_ and NiCl_2_ dual-component mixed solution. To this, 100 mL of freshly prepared DMAB (0.5 M) was added. Then, 5 mL of KOH (1 M) was slowly dropped into the mixed solution. After continuous stirring, the mixed solution was sealed and stored in the dark for 48 h incubation, which yielded a yellow hydrogel. This hydrogel was thoroughly washed and dried, successfully yielding the aerogel sample. To investigate the effect of KOH on the aerogel properties, a series of samples were prepared under controlled conditions with varying amounts of 1 M KOH solution, namely 0, 1, 3, 5, and 7 mL; these samples were labeled as 0 K–FeNi, 1 K–FeNi, 3 K–FeNi, 5 K–FeNi, and 7 K–FeNi, respectively. Additionally, single-component Fe-based and Ni-based aerogels were prepared: 5 K–Fe and 5 K–Ni. Figure 1 shows the experimental preparation process.

### 2.3. Sample Characterization

The morphological features of the aerogels were characterized via scanning electron microscopy (SEM, ZEISS Gemini SEM 300, Oberkochen, Germany) and transmission electron microscopy (TEM, FEI Talos F200x, Hillsboro, OR, USA). Their morphology and elemental composition were analyzed via energy dispersive X-ray (EDX) spectroscopy. TEM, high-resolution TEM (HRTEM), and high-angle annular dark-field scanning transmission electron microscopy (HAADF-STEM) images; selected area electron diffraction (SAED) patterns; and energy dispersive X-ray spectra (EDS) and elemental mapping images were obtained at an accelerating voltage of 200 kV using the FEI Talos F200x transmission electron microscope. The phase structure was detected using a Rigaku (Tokyo, Japan) ultima IV powder X-ray diffractometer (XRD, Cu Kα1). X-ray photoelectron spectroscopy (XPS) was performed on the aerogel surface using Thermo scientific (Waltham, MA, USA) K-Alpha to characterize the elemental electronic states. Micro Raman spectroscopy was performed at a laser wavelength of 514 nm with Horiba (Irvine, CA, USA) LabRAM HR evolution to analyze the chemical bonding in the aerogel samples. The FT-IR infrared spectra of the samples were obtained using Thermo scientific Nicolet iS5. The electron spin resonance (ESR) properties were characterized using a Bruker (Billerica, MA, USA) EMX-plus-6/1 electron paramagnetic resonance spectrometer with measurements conducted at a temperature of 25 °C, utilizing a quartz tube with a diameter of 4.19 mm.

### 2.4. Electrochemical Measurements

Electrochemical testing was performed on the aerogel samples on an electrochemical workstation in a typical three-electrode mode. A carbon paper coated with a catalyst ink was used as the working electrode (0.5 cm × 0.5 cm; coverage area: 0.25 cm^2^); a Hg/HgO electrode and Pt wire electrode were chosen as the reference and counter electrodes, respectively. A total of 5 mg of the sample, 20 µL of Nafion solution, 330 µL of ethanol, and 650 µL of deionized water were mixed and subjected to intensive ultrasonic treatment for 90 min, resulting in a uniformly dispersed catalyst ink (5 mg mL^−1^). The prepared catalyst ink, 16.3 µL, was evenly dropped on the carbon paper with a loading amount of 0.33 mg cm^−2^ and dried under an infrared lamp before testing. The standard potential of the sample was converted to the reversible hydrogen electrode (RHE) using the formula E (RHE) = E (Hg/HgO) + 0.059 × pH + 0.098. Linear sweep voltammetry (LSV) and cyclic voltammetry (CV) curves were measured at scan rates of 10 and 10 mV s^−1^, respectively. The overpotential η was calculated using the following formula: η (V) = E (RHE) −1.23 V. All the LSV electrochemical data were displayed after IR correction. The Tafel slope was obtained using the formula η = a + b log j, where a is the exchange current density, b is the Tafel slope, and j is the current density. After 5000 cycles of CV at a scan rate of 100 mV s^−1^, electrochemical impedance spectroscopy (EIS) spectra were recorded at a standard electrode potential of 1.47 V and frequency range of 0.1–1000 kHz. A 12 h stability test was conducted using the potentiostatic polarization method at a standard electrode voltage of 1.47 V.

## 3. Results and Discussion

**Figure 2:**
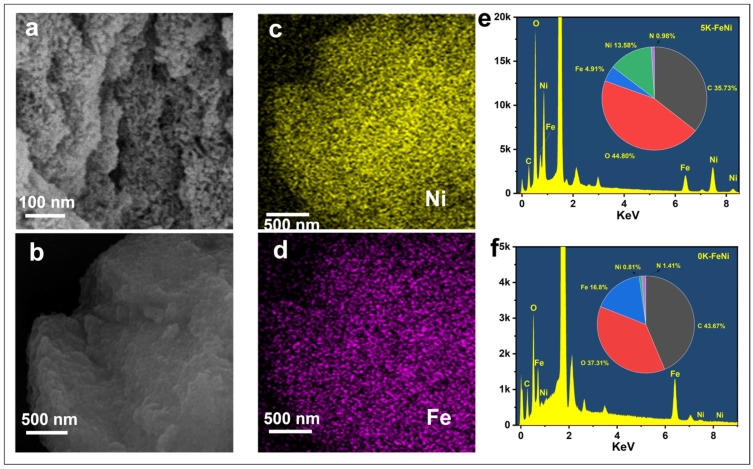
(**a**) SEM image of 5 K–FeNi. (**b**–**d**) EDX element mapping images. (**e**) EDX spectrum of 5 K–FeNi (quantitative value/atom%: O 44.80, C 35.73, Ni 13.58, Fe 4.91, and N 0.98). (**f**) EDX spectrum of 0 K–FeNi (quantitative value/atom%: O 37.31, C 43.67, Ni 0.81, Fe 16.8, and N 1.41).

**Figure 3:**
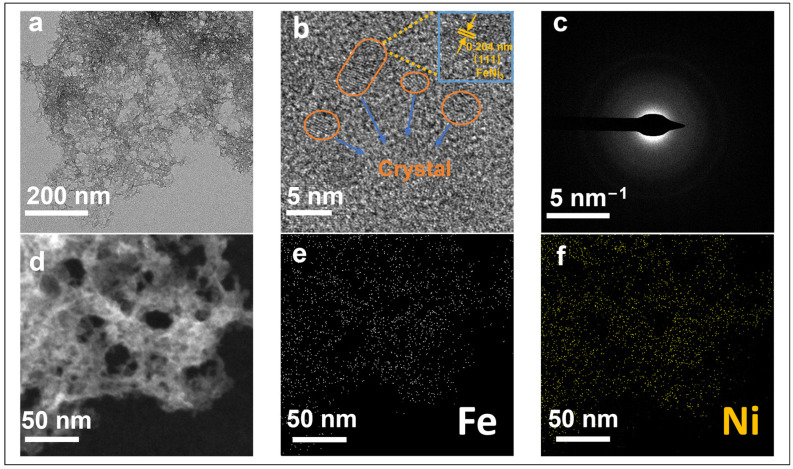
(**a**) TEM image; (**b**) HRTEM image; (**c**) SAED pattern HAADF-STEM micrograph; and (**d**) HAADF-STEM image of 5 K-FeNi and (**e**,**f**) EDX mapping of Fe and Ni in 5 K–FeNi.

The 5 K–FeNi aerogel samples were characterized via SEM, TEM, and EDX analyses; the corresponding results are shown in Figure 2 and Figure 3. Figure 2a shows that numerous interconnected nanoparticles together create the aerogel nanoporous structure of the 5 K–FeNi aerogel samples, indicating the successful synthesis of a 3D porous FeNi-based aerogel. The distribution of Fe and Ni in the aerogel samples was analyzed via EDX spectroscopy, which revealed that these elements were uniformly distributed throughout the sample (Figure 2c,d). Figure 2e shows that the Ni-to-Fe molar ratio in the 5 K–FeNi sample is approximately 2.76:1, close to the designed Ni-to-Fe molar ratio of 2.5:1. In contrast, Figure 2f shows that the Ni-to-Fe molar ratio in the 0 K–FeNi sample deviates significantly from the designed ratio. This suggests that the introduction of OH^−^ ions enhances the Ni content in the aerogel, which considerably influences the regulation of Ni content.

Figure 3a shows the TEM results of the 5 K–FeNi aerogel sample, highlighting its honeycomb-like 3D porous structure. Figure 3b shows the HRTEM image of the 5 K–FeNi aerogel, wherein large amorphous regions and smaller areas of long-range ordered nanoclusters can be observed along with nanoclusters embedded in the amorphous areas. The nanoclusters and amorphous areas are closely interconnected, so the sample mainly comprises an amorphous structure, with small crystallites of <5 nm and an interplanar spacing of ~0.204 nm, corresponding to the (111) planes of FeNi_3_. The HAADF-STEM electron diffraction pattern in the SAED mode (Figure 3c) shows only a single diffraction spot, with no apparent diffraction rings or spots; this further confirms that the 5 K–FeNi aerogel sample is primarily amorphous. The corresponding elemental mapping images in Figure 3e,f show that Fe and Ni are uniformly distributed throughout the aerogel sample.

**Figure 4:**
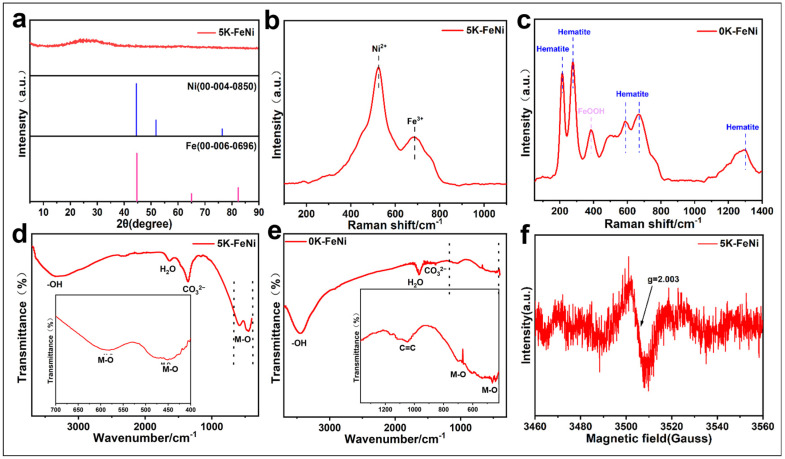
(**a**) XRD pattern of 5 K–FeNi; (**b**) Raman spectra of 0 K–FeNi; (**c**) Raman spectra of 5 K–FeNi; (**d**) FT-IR spectra of 5 K–FeNi; (**e**) FT-IR spectra of 0 K–FeNi; and (**f**) EPR spectra of 5 K–FeNi.

The phase characteristics of the 5 K–FeNi bimetallic aerogel sample were analyzed via XRD analysis. The XRD spectrum in Figure 4a does not show any distinct diffraction peaks characteristic of the FeNi alloy or Fe and Ni; only a very weak bulge appears near the 2-theta diffraction angle of 26°. Combined with the SAED pattern and HAADF-STEM micrograph in Figure 3c, this finding indicates that the 5 K–FeNi bimetallic aerogel particles have very low crystallinity, which confirms their amorphous structure. Figure 4b,c show the Raman spectra for 5 K–FeNi and 0 K–FeNi aerogel samples. The peaks at 524 and 680 cm^−1^ for the 5 K–FeNi sample are attributed to the Ni^2+^ and Fe^3+^ vibrational peaks, respectively [28,29]. In contrast, the 0 K–FeNi sample does not show any significant Ni vibrational peaks, with the main peaks appearing at 214, 277, 385, 587, 666, and 1292 cm^−1^. The peaks at 214, 277, 587, 666, and 1292 cm^−1^ are associated with the vibrational peaks of hematite-type iron, whereas that at 385 cm^−1^ is attributed to FeOOH [30,31,32]. The FT-IR spectra of 0 K–FeNi and 5 K–FeNi samples (Figure 4d,e) show distinct absorption peaks near 3328, 1633, 1357, 581, and 451 cm^−1^ for the latter. The absorption peak at 3328 cm^−1^ corresponds to the stretching vibration of the hydroxyl group that at 1633 cm^−1^ corresponds to the deformation vibration of water molecules, and that near 1359 cm^−1^ corresponds the CO_3_^2−^ vibration. The absorption peaks below 800 cm^−1^ are attributed to M-O vibration [33,34,35]. The ESR detection technique was used to measure the unpaired electrons in the 5 K–FeNi sample. Figure 4f shows a distinct ESR signal at g = 2.003, indicating the presence of a certain number of oxygen vacancies in the sample [36].

**Figure 5:**
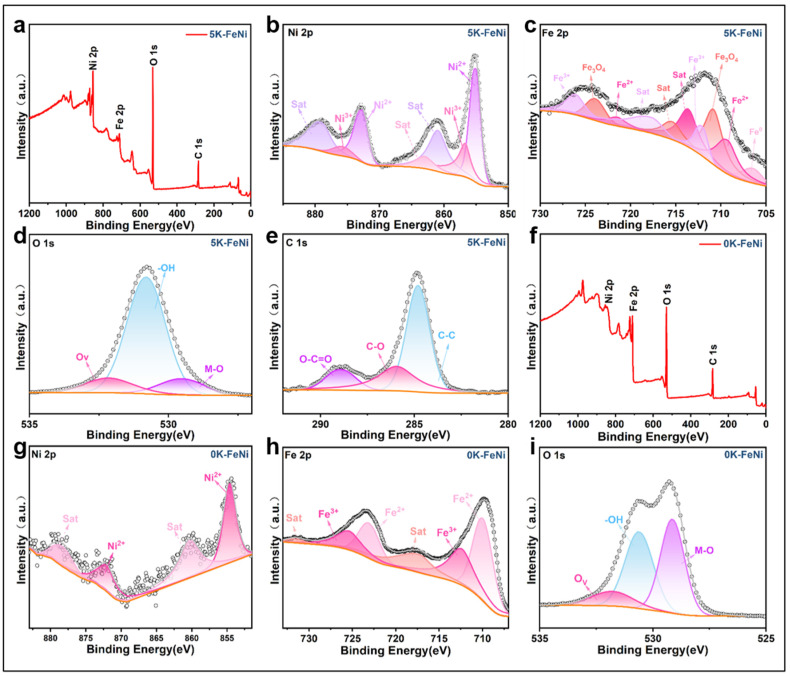
(**a**–**e**) XPS spectra and Fe 2p, Ni 2p, O 1s, and C1s core spectra of the 5 K–FeNi sample. (**f**–**i**) XPS spectra and Fe 2p, Ni 2p, and O 1 s core spectra of the 0 K–FeNi sample.

The chemical states of surface elements and electronic structures of 5 K–FeNi and 0 K–FeNi samples were determined via XPS. The full XPS spectrum in Figure 5a shows that the samples contain high amounts of Fe and Ni, consistent with the EDS results. Subsequent analyses of the XPS fine spectra of Fe, Ni, C, and O were performed. The high-resolution spectrum of Ni in Figure 5b reveals that it primarily exists as Ni^2+^ and Ni^3+^ in the aerogel samples. The peaks at 855.06 and 872.68 eV correspond to Ni^2+^ 2p3/2 and Ni^2+^ 2p1/2, respectively, with a satellite peak appearing at 860.95 eV [37]. Those at 856.70 and 875.76 eV are attributed to Ni^3+^ 2p3/2 and Ni^3+^ 2p1/2, with satellite peaks appearing at 863.02 and 879.07 eV, respectively [38]. The high-resolution spectrum of Fe in Figure 5c indicates that it primarily exists as Fe^0^, Fe^2+^, Fe^3+^, and Fe_3_O_4_. The peak at 706.49 eV corresponds to Fe^0^ 2p3/2 [39]; those at 709.26 and 721.5 eV belong to Fe^2+^ 2p3/2 and Fe^2+^ 2p1/2, respectively, with a significant satellite peak at 713.62 eV. The peaks at 710.82 and 723.92 eV belong to Fe_3_O_4_ 2p3/2 and Fe^2+^ 2p1/2 [40], respectively, with a clear satellite peak at 715.35 eV. Additionally, those at 712.06 and 726.12 eV belong to Fe^3+^ 2p3/2 and Fe^3+^ 2p1/2, respectively with a satellite peak at 717.72 eV [41,42]. The high-resolution spectrum of O in Figure 5d shows that the 5 K–FeNi sample contains M-O, -OH, and Ov, corresponding to the peaks at 529.52, 530.80, and 532.15 eV [36]. The high-resolution spectrum of C in Figure 5e reveals that carbon primarily exists as C-C, C-O, and O-C=O, with their vibrational peaks occurring at 284.12, 284.8, 285.5, 285.94, and 288.39 eV [43,44,45]. XPS analysis was also performed on the 0 K–FeNi sample, wherein Ni primarily existed as Ni^2+^ and Fe as Fe^2+^ and Fe^3+^. The forms of carbon and oxygen were the same as in the 5 K–FeNi sample. A comparison of the full XPS spectra of 0 K–FeNi (Figure 5f) and 5 K–FeNi (Figure 5a) showed that the 5 K–FeNi sample contained significantly higher amounts of Ni than Fe due to the introduction of the hydroxyl group. Thus, the Ni-to-Fe molar ratio conforms with the experimental design ratio. The fine XPS spectrum of Ni in Figure 5g shows a clear presence of Ni^2+^ in the 0 K–FeNi samples without any significant peaks for Ni^3+^. The fine XPS spectrum of Fe in Figure 5h shows characteristic peaks for Fe^2+^ and Fe^3+^ in the 0 K–FeNi sample, with no significant peaks for Fe^0^. This finding suggests that a relatively weak synergistic effect exists between Ni and Fe in the 0 K–FeNi sample. The fine XPS spectra in Figure 5d,i indicate the presence of oxygen vacancies, consistent with the ESR test results.

**Figure 6:**
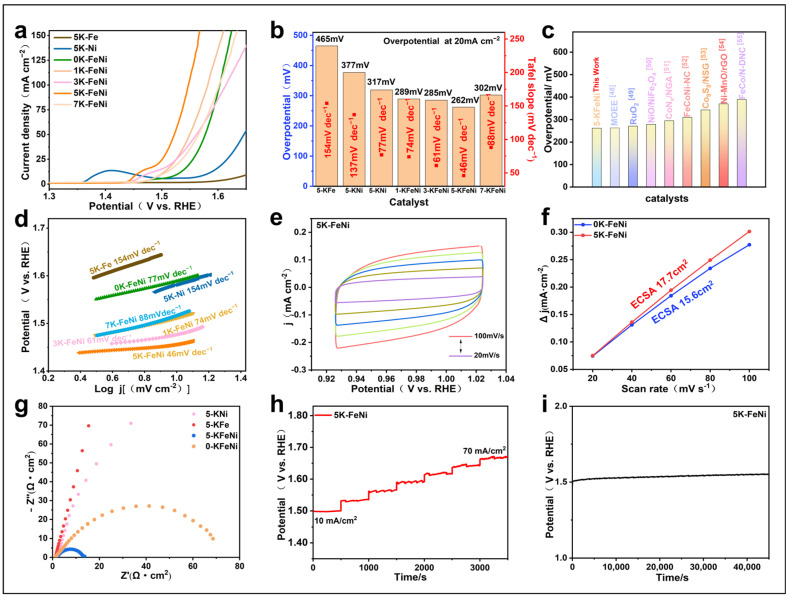
OER measurements in 1 M KOH. (**a**) OER polarization curves for various catalysts at a scan rate of 10 mV s^−1^. (**b**) Overpotentials at 20 mA cm^−2^ and Tafel slopes for all the prepared catalysts. (**c**) A comparison of the overpotential of as-prepared catalysts at a current density of 20 mA cm^−2^ with that of other advanced OER catalysts at a current density of 10 mA cm^−2^ [48,49,50,51,52,53,54,55]. (**d**) Tafel plots derived from the OER polarization curves in (**a**). (**e**) CV curves of 5 K–FeNi (20–100 mV s^−1^). (**f**) Cdl was calculated from the capacitive current density (Δj/2 = (ja − jc)/2) vs. scan rate of all the as-prepared samples. (**g**) Nyquist plots for all the as-prepared samples. (**h**) Multistep chronopotentiometry curve of 5 K–FeNi. (**i**) Long-term durability tests at a constant current density of 20 mA cm^−2^.

The LSV curves in Figure 6a demonstrate that the aerogel samples prepared using different KOH amounts exhibit considerably different electrochemical activities. Figure 6a,b show that the electrocatalytic activity initially increases and then decreases with increasing KOH content. Among them, the 5 K–FeNi sample exhibits the lowest overpotential of 262 mV at a current density of 20 mA cm^−2^. Moreover, the electrocatalytic activity of the Fe/Ni-based aerogel samples prepared in the KOH system is superior to that of the 0 K–FeNi sample prepared without KOH (317 mV), as well as Fe and Ni (5 K–Fe (465 mV), 5 K–Ni (377 mV), and 0 K–FeNi (366 mV). They also outperform other catalysts such as commercial RuO_2_ at a current density of 10 mA cm^−2^ and an overpotential of 271 mV (Figure 6c). This suggests that adding appropriate amounts of KOH can facilitate the formation of Fe/Ni-based aerogels and that the synergistic effect of Fe and Ni can enhance the catalytic activity of aerogels, thereby reducing the overpotential of the OER. A comparison of the XPS spectra of O in the 0 K–FeNi and 5 K–FeNi samples (Figure 5d,i) shows that 5 K–FeNi contains a higher proportion of M-OH, indicating a relatively high amount of Fe/Ni-based hydroxides. The interface of Fe/Ni-based hydroxides and FeNi alloy promotes *OH adsorption and *OOH decomposition in the OER [46]. The LSV curves also show a distinct oxidation peak at 1.45 V for the 5 K–FeNi sample, corresponding to high-valence Ni oxidation [47]; however, no Ni oxidation peak is observed for the 0 K–FeNi samples. This suggests that only a small amount of Ni was added during 0 K–FeNi synthesis; therefore, a synergistic effect with Fe was not effectively achieved.

The Tafel slope plot obtained from the LSV curves (Figure 6d) further reveals the reaction kinetics of different aerogel samples. The Tafel slopes for 0 K–FeNi, 1 K–FeNi, 3 K–FeNi, and 7 K–FeNi are 77, 74, 61, and 88 mV dec^−1^, respectively. However, that for 5 K–FeNi was only 46 mV dec^−1^, indicative of its higher OER activity. Thus, the introduction of KOH effectively regulated the ratios of Ni and Fe in the Fe/Ni-based aerogels, considerably affecting their catalytic performance.

CV measurements on the 5 K–FeNi aerogel sample were conducted at different scan rates (Figure 6e). The electrochemical active surface area (ECSA) was used to indirectly analyze the electrocatalytic activity of all the samples. A larger ECSA indicates more exposed active sites, which in turn suggests higher catalytic activity [56]. The ECSA of the samples was determined using the formula ECSA = C_dl_/Cs, where Cs is the double-layer capacitance per unit area. Cdl was calculated from the CV curves at different scan rates (Figure 6e). Figure 6f shows that the Cdl of 5 K–FeNi is 0.7075 mF, higher than that of 0 K–FeNi (0.625 mF). The calculated ECSA for 5 K–FeNi was 17.7 cm^2^, greater than that of the 0 K–FeNi sample (15.6 cm^2^).

The charge transfer kinetics of catalysts during the OER were determined via EIS. As shown in Figure 6g, the Rct of the 5 K–FeNi aerogel sample is 13 Ω cm^2^, considerably lower than that of the 0 K–FeNi (77 Ω cm^2^), 5–KFe (1133 Ω cm^2^), and 5–KNi (305 Ω cm^2^) samples. Research has shown that higher valent Ni (Ni^3+^/Ni^4+^) is of great significance for the oxygen evolution reaction. When synthesizing aerogels, the valency of Ni was primarily 2+. Upon adding Fe, Ni^2+^ further oxidized to become Ni^3+^ or Ni^4+^ with higher valent states, which enhanced the electron migration efficiency [57].

Electrochemical stability is an important parameter for catalyst materials. Figure 6h shows the multistep chronopotentiometry curve for the 5 K–FeNi sample, indicating that the voltage remains stable within a small range for 500 s at a current density of 10 mA cm^−2^; similar behavior was observed at higher current densities. The long-term chronopotential stability test results in Figure 6i indicate that the voltage of the 5 K–FeNi sample remains relatively unchanged over a 12 h testing period, demonstrating its excellent OER stability. Compared with the commercial noble metal catalyst RuO_2_, 5 K–FeNi not only exhibited lower overpotential but also benefited from the easy availability of Ni and Fe as well as its long-term stability. Thus, it is a highly promising, efficient, and cost-effective OER catalyst.

The 5 K–FeNi catalyst prepared in an alkaline system exhibited excellent OER catalytic performance. This was primarily attributed to the synergistic effect between Fe and Ni, which accelerated the OER kinetics. Fe induced the evolution of Ni^3+^ to Ni^4+^ during the OER, which increased the valence state of Ni and enhanced the electron transfer efficiency between Ni and Fe. Additionally, an increase in oxygen vacancies altered the electronic structure of the material, thereby increasing the number of catalytic active sites and enhancing the adsorption and desorption of intermediates during the OER. Moreover, the OER rate of the catalyst increased [58,59]. The 5 K–FeNi sample also had a unique crystalline–amorphous heterostructure, wherein an irregular atomic arrangement of the amorphous structure provided more active sites for the OER. During the catalytic process, the amorphous structure changed its morphology and adjusted its atomic arrangement to accelerate the electron transfer process. This was conducive to improving the catalytic activity and surface reactivity of the catalyst.

## 4. Conclusions

Herein, Fe/Ni-based aerogel catalysts were effectively synthesized via wet chemical reduction coprecipitation. They had a 3D porous network structure formed by fine FeNi-based nanoparticles. The nanoclusters were embedded in amorphous areas, effectively forming a closely interconnected crystalline–amorphous heterostructure. The multiscale nanostructure, 3D porous structure, and crystalline–amorphous heterointerface structure of the Fe/Ni-based aerogels provided more reactive sites and a larger driving force for electron migration. The electronic coupling synergistic effect between Fe and Ni enhanced the electron transfer efficiency between oxygen and hydrogen ions in an aqueous solution, thereby accelerating the OER kinetics. The Fe/Ni-based amorphous aerogel catalyst synthesized from non-noble metals demonstrated excellent OER performance and prolonged stability, rendering it a highly promising, high-efficiency, and low-cost OER catalyst.

## Data Availability

Data are contained within the article.
